# 5′HS5 of the Human *β-globin* Locus Control Region Is Dispensable for the Formation of the *β-globin* Active Chromatin Hub

**DOI:** 10.1371/journal.pone.0002134

**Published:** 2008-05-07

**Authors:** Ping Kei Chan, Albert Wai, Sjaak Philipsen, Kian-Cheng Tan-Un

**Affiliations:** 1 Department of Zoology, Kadoorie Biological Science Building, The University of Hong Kong, Hong Kong Special Administrative Region (SAR), China; 2 Erasmus MC, Department of Cell Biology, Rotterdam, The Netherlands; University of Munich and Center of Integrated Protein Science, Germany

## Abstract

Hypersensitive site 5 (5′HS5) of the *β-globin* Locus Control Region functions as a developmental stage-specific border in erythroid cells. Here, we have analyzed the role of 5′HS5 in the three dimensional organization of the *β-gene* locus using the Chromatin Conformation Capture (3C) technique. The results show that when 5′HS5 is deleted from the locus, both remote and internal regulatory elements are still able to interact with each other in a three-dimensional configuration termed the Active Chromatin Hub. Thus, the absence of 5′HS5 does not have an appreciable effect on the three dimensional organization of the *β-globin* locus. This rules out models in which 5′HS5 nucleates interactions with remote and/or internal regulatory elements. We also determined the binding of CTCF, the only defined insulator protein in mammalian cells, to 5′HS5 by using chromatin immunoprecipitation (ChIP) assays. We detect low levels of CTCF binding to 5′HS5 in primitive erythroid cells, in which it functions as a border element. Surprisingly, we also observe binding levels of CTCF to 5′HS5 in definitive erythroid cells. Thus, binding of CTCF to 5′HS5 *per se* does not render it a functional border element. This is consistent with the previous data suggesting that CTCF has dual functionality.

## Introduction

The human *β-globin* locus contains five genes that are arranged in the same order as their developmental expression pattern: 5′-ε (embryonic) Gγ Aγ (foetal) δ β (adult)-3′. The major activating region, termed the Locus Control Region (LCR), is located 6 to 22 kb 5′ to the ε-globin gene. The most prominent charactistic of the human *β-globin* LCR is that it drives high level, tissue-specific, copy number-dependent and position-independent expression of linked transgenes in mice [1]. The core components of the LCR are the DNase I hypersensitive sites (5′HS1-5) [2]. Each of the hypersensitive sites holds a unique array of transcription factor binding sites, including those for GATA1, NF-E2 and EKLF proteins. This suggests that the HS in the human LCR may have different stage-specific activities and cannot be replaced functionally by each other [3], [4]. Transcriptional activation of the *β-like globin* genes is thought to be a multi-step system. This model includes the active involvement of erythroid transcription factors in the spatial organization of the locus, and increased accessibility to trans-acting factors by recruitment of chromatin remodeling complexes [5]. The resultant transcriptionally active locus is arranged in a three dimensional structure termed the active chromatin hub (ACH), in which the LCR hypersensitive sites interact directly with the transcribed genes through a looping mechanism [6], [7]. The deduced spatial arrangement of the regulatory elements and the genes in the ACH offers an explanation for the observation that a gene proximal to the LCR has a transcriptional advantage over a more distally located gene [8], [9]. The mode of action of the LCR to the *β-globin* genes appears to be orientation dependent as an inverted LCR is incapable of activating downstream globin genes at high levels, and the ε-gene can not be activated when placed upstream of the LCR [10]. This property of the LCR may be due to the spatial organization of its hypersensitive sites, but it is also possible that the locus contains elements that block LCR action in one of the directions [11].

Recently, we have shown that human 5′HS5 acts as a developmental stage-specific border element in erythroid cells. In that study, we placed a marked *β-globin* gene upstream of the LCR and the genes, using PAC constructs of the human *β-globin* locus in which 5′HS5 was flanked by loxP sites [11].

In the present study, we applied Chromosome Conformation Capture (3C) technology to further investigate the functional properties of 5′HS5 *in vivo*. The rationale behind the use of the 3C technique was to delineate the spatial organization of the *β-globin* locus upon Cre/loxP-mediated deletion of 5′HS5 in human *β-globin* transgenes in mice. To date, directionality of the LCR has not yet been studied using the 3C technique in the context of the three-dimensional structure of the human *β-globin* locus. 3C analysis of the human *β-globin* transgenes was performed at the primitive and definitive stages of erythropoiesis. Since 5′HS5 functions as a border in primitive erythroid cells, any major alterations in the 3D organization of the locus related to this property should be revealed. We also performed 3C analysis in the same transgenic lines in which 5′HS5 had been removed by Cre-mediated excision.

The first insulator element reported in vertebrates is 5′HS4 (cHS4) in the chicken *β-globin* locus [12]. Binding of the 11 Zn-finger protein CTCF to the FII region of the cHS4 is thought to play an important role in the activity of cHS4 [13], [14]. To investigate the potential activity of CTCF as an enhancer blocking protein on human 5′HS5 *in vivo*, we performed chromatin immunoprecipitation assays using antibodies directed against CTCF.

## Results

### PAC constructs for 3C analysis of the human *β-globin* locus

**Figure 1:**
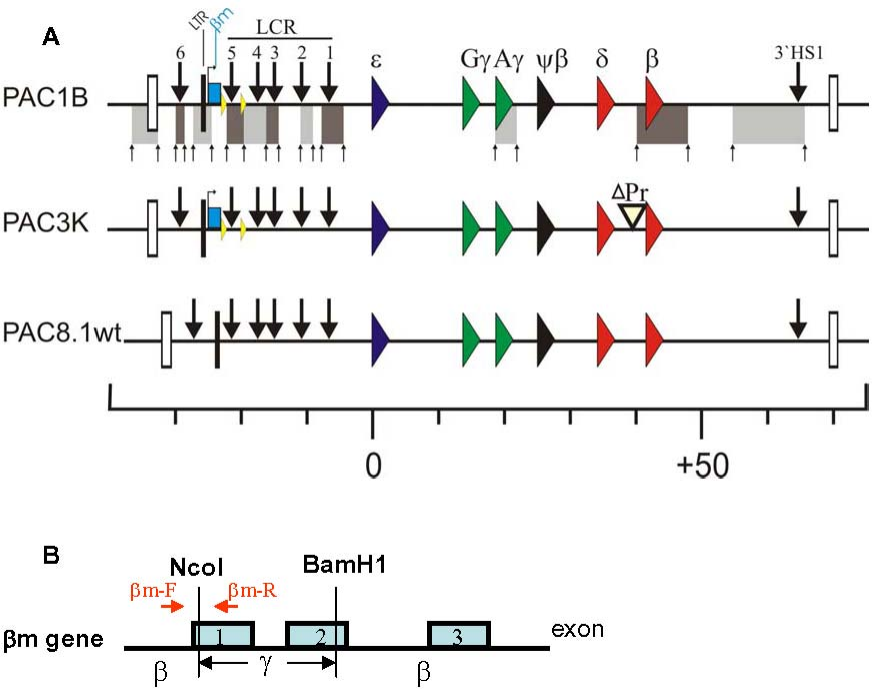
Schematic presentation of the human *β-globin* locus in PAC transgenic constructs. A) Arrows on top of the locus depict the individual hypersensitive sites within the LCR. *β-like globin* genes are indicated by large triangles with different colors. The *β-mark globin* gene was indicated by light blue rectangle with *β*m labeled on top of it. LTR located at the 5′-upstream of the *β*m gene was indicated by the small rectangle with black in color. The HS5 is flanked by loxP sites indicated by small triangle on the locus. PACΔ constructs were generated by homologous recombination according to Iman *et al*., 2000. The white boxes indicate the *olfactory receptor* (OR) genes which flank the *β-globin* locus. HindIII restriction sites (small arrows) and the DNA fragments used in the 3C analysis (solid rectangles) are shown below the PAC1B locus. Distances are in kb counting from the transcription initiation site of the ε*-globin* gene. B) Structure of the marked *β*-globin gene (*β*m) and the ChIP-*β*m primers location.

To examine the potential conformational changes of the ACH in the presence or absence of 5′HS5 in the context of the human *β-globin* locus, we employed transgenic mice carrying modified human β-globin PAC transgenes (Fig. 1, [11]). The transgenes were derived from PAC185 which contains the entire human *β-globin* gene cluster. Homologous recombination in *E. coli* DH10B cells [15] was used for generation of the constructs. To determine the effect of deletion of 5′HS5 on the three dimensional (3D) organization of the locus, 5′HS5 was flanked by loxP sites to enable Cre-mediated deletion of 5′HS5 (PACΔ1B). The basal promoter and 5′ untranslated regions of the β-globin gene, from −139 to +49 relative to the cap site of the gene, has been removed to generate the *β-globin* promoter deletion (PAC3K; Fig. 1). Intactness of the transgenes was carefully mapped by Southern blot analysis using a total of 11 different restriction enzymes and hybridization with cosmid LCRε, -γγδβ probes [16], and smaller probes along the *β-globin* locus [11].

### 3C analysis of the PAC constructs with the βm gene and 5′HS5 flanked by loxP sites

**Figure 2:**
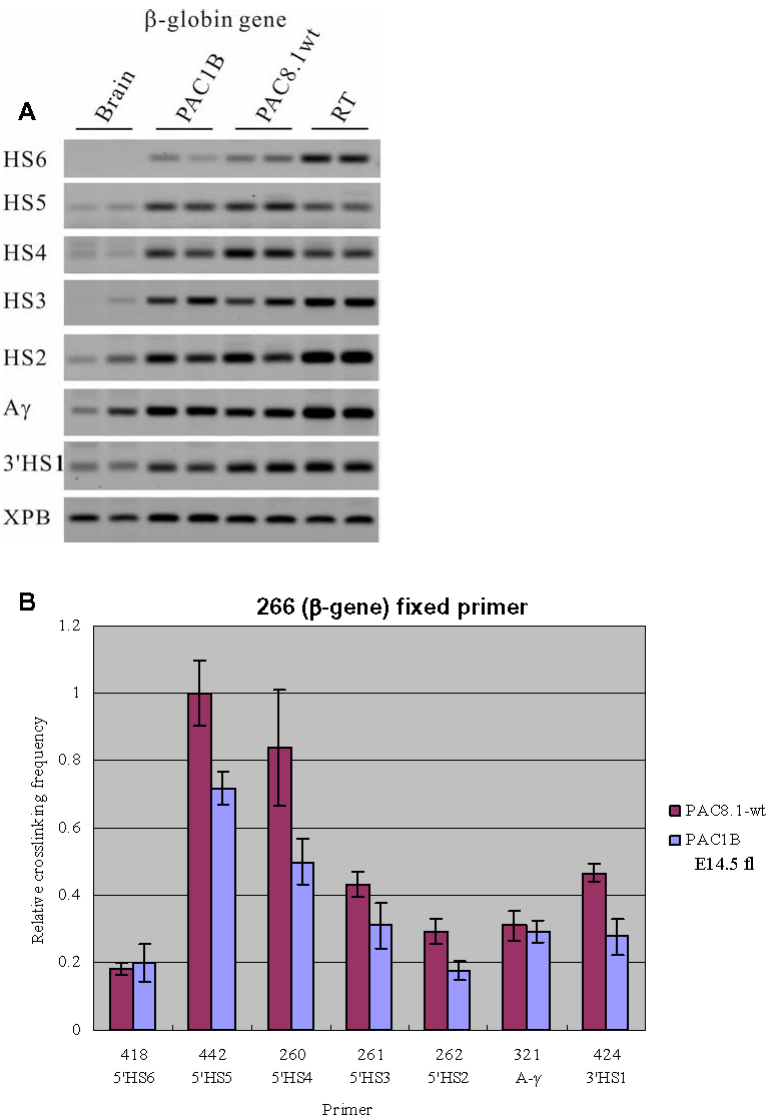
3C analysis of the PAC1B transgene using a primer from the human *β-globin* gene in combination with primers from other parts of the locus. Fetal livers were collected from E14.5 embryos for this set of 3C experiments. (A) Representative examples of the PCR fragments resulting from the 3C experiments. WT: PAC8.1wt; RT: Random template control. (B) Histogram of the relative crosslinking efficiencies after quantitation and normalization. The histograms are the average of at least three separate experiments, with each PCR performed in triplicate.

Previous studies on human *β-globin* transgenes in mice have shown that the *β-globin* gene interacts with the ACH when it is transcribed in definitive erythroid cells [7]. In these studies, the Chromosome Conformation Capture (3C) technique [6], [7], [17] was used to examine the three dimensional organization of the *β-globin* locus. First, we determined whether introduction of the *β-marked* (*βm*) gene, which is an artificially modified β-globin gene with part of its exon 2 replaced by equivalent Aγ-globin gene [9] (Fig. 1B), and flanking 5′HS5 by loxP sites *per se* caused conformational changes of the *β-globin* locus. We performed 3C analysis on the PAC transgenes and compared this directly to the results obtained with the wild type locus (PAC8.1; Fig. 2). In the wild type locus, 5′HS1-5 of the LCR and the HS in the 3′ flanking region (3′HS1) interact with each other to form the ACH of the *β-globin* locus. The results of 3C analysis on the PAC1B transgene show that the spatial structure of the locus is basically unaltered when compared with that of wild type PAC8.1. In brain, a non-erythroid tissue, the PAC transgene shows an essentially linear conformation of the *β-globin* locus (data not shown). This is consistent with the notion that the formation of ACH is tissue specific [6].

### Effect of the deletion of the 5′HS5 on ACH formation

To study the effect of 5′HS5 on the spatial organization of the *β-globin* locus, we compared the PAC1B line with the PACΔ1B line, in which 5′HS5 had been deleted by the action of Cre recombinase [15].

It has been proposed that transcriptional activation of *β-like globin* genes at each stage of development is a multi-step process [5]. An LCR holocomplex is formed to allow the accessibility of transcription and chromatin remodeling factors to the locus, thus providing a high local concentration of the relevant trans-acting factors for efficient transcription. The resultant functional active chromatin hub (ACH) comprises the LCR holocomplex interacting directly with the transcribed genes [6], [7], [18]. This model implies that maintaining the integrity of the ACH is the key to create a chromatin domain permissive for transcriptional regulation.

**Figure 3:**
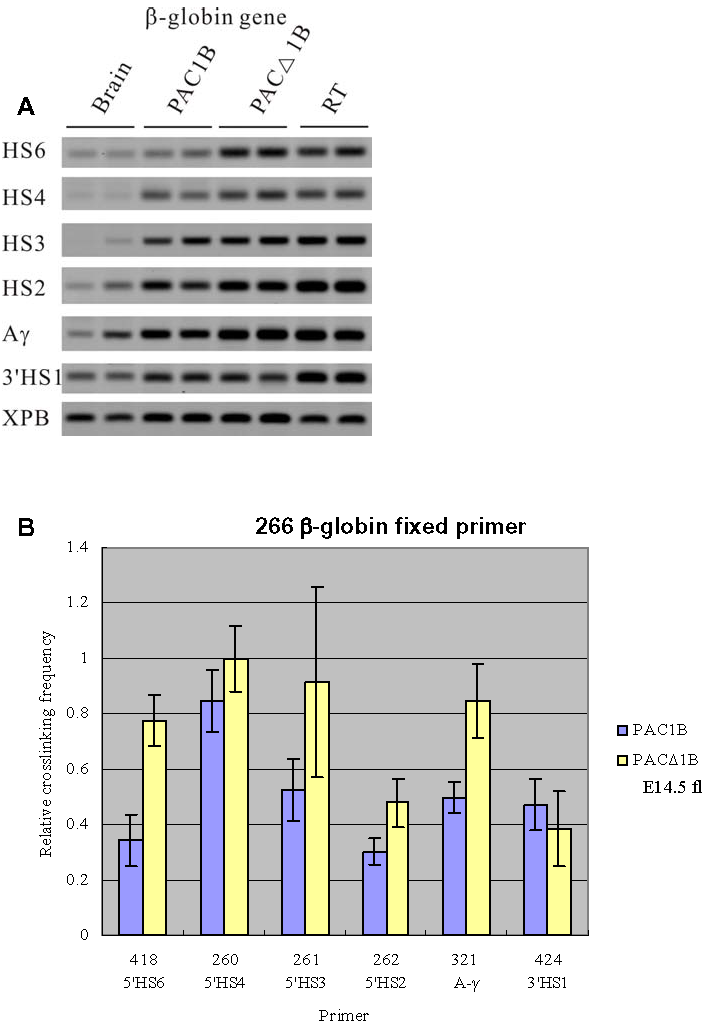
3C analysis of the PAC1B and PACΔ1B transgenic lines using a primer from the human *β-globin* gene in combination with primers from other parts of the locus. Fetal livers were collected from E14.5 embryos for this set of 3C experiments. (A) Representative examples of the PCR fragments resulting from the 3C experiments. (B) Histogram of the relative crosslinking efficiencies after quantitation and normalization. The histograms are the average of at least three separate experiments, with each PCR performed in triplicate.

To examine the possible consequences of deletion of 5′HS5 on ACH formation, we compared the 3D structures of the PAC1B and PACΔ1B transgenes in definitive erythroid cells isolated from 14.5 dpc fetal livers with the 3C technique (Fig. 3). The results show that the overall 3D of the *β-globin* locus has remained the same when 5′HS5 is deleted (PACΔ1B). However, the relative crosslinking frequency of the 5′HS6, the distal hypersensitive site located about 6 kb 5′ upstream to the HS5 [19], with the *β-globin* gene is increased by upon the removal of 5′HS5 (Fig. 3B, primer 418). This increase in association frequency with the ACH is presumably due to the closer proximity of 5′HS6 to the LCR after Cre-mediated excision of 5′HS5. 5′HS6 is located 800bp closer to the LCR in the PACΔ1B line. Besides, in the absent of HS5, the β-gene promoter interacts strongly with the A-γ promoter.

In mammals, two developmental switches of hematopoiesis occur during ontogeny. In transgenic mice carrying the human β-globin locus, yolk sac-derived primitive erythroid cells express the human embryonic ε- and fetal γ-globin genes [16]. β-like globin gene expression switches to the fetal γ- and adult δ- and β-globin genes during early definitive erythropoiesis between E12.5 and E14.5. The LCR plays an important role in the regulation of expression of the β-like globins, and the order and distance of the genes relative to the LCR are important parameters for the developmental switch [8], [9], [20]. These developmental switches of hematopoiesis are accompanied by changes in chromatin structure and spatial organization of regulatory elements throughout the locus [6], [7], [21]. In a previous study with the human β-globin PAC transgenes we have shown that 5′HS5 functions as an enhancer blocker in embryonic erythroid cells [11]. Therefore, it is interesting to determine the possible conformational changes of the locus without 5′HS5 in embryonic erythroid cells.

PAC3K transgenic lines, in which the promoter of the β-globin gene in the normal position is deleted, have been used for the 3C analysis. The rationale behind the usage of the PAC3K construct for performing the 3C analysis is that in this configuration, the *βm* gene located upstream of the LCR is maximally activated in definitive cells, while expression in primitive cells is only detectable after deletion of 5′HS5. We therefore compared the locus-wide crosslinking frequencies of the PAC3K and PACΔ3K (PAC construct with deletion of 5′HS5) transgenes at different developmental stages. In the fetal liver, we observed that the 3D conformation of the PAC3K and PACΔ3K transgenes is virtually identical to that observed for the PAC1B and PACΔ1B transgenes (data not shown). This implies that neither 5′HS5 nor the β-globin promoter is required for structural changes in the ACH occurring during the process of β-globin gene switching [7], [18], [22]. The latter is in agreement with Patrinos *et al*. [23] who have shown that deletion of the β-globin promoter alone is not sufficient to alter the 3D configuration of the ACH.

**Figure 4:**
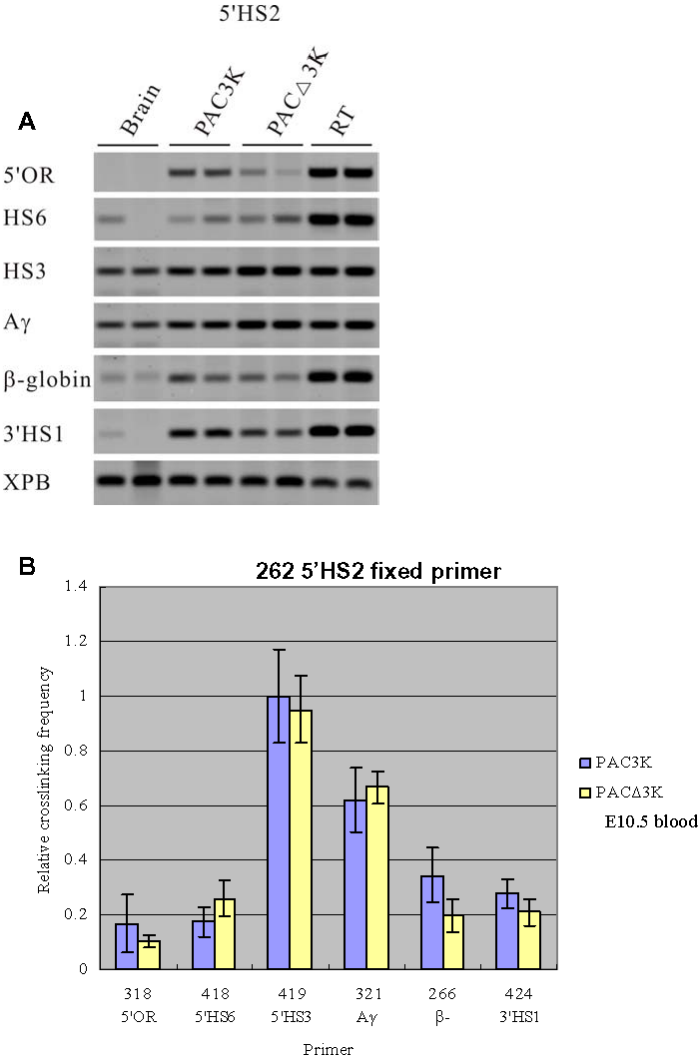
3C analysis of the PAC3K and PACΔ3K transgenes using a primer from 5′HS2 as the fixed primer. Embryonic blood was collected from E10.5 embryos for this set of 3C experiments. (A) Representative examples of the PCR fragments resulting from the 3C experiments. RT: Random template control. (B) Histogram of the relative crosslinking efficiencies after quantitation and normalization.

Since 5′HS5 functions as a border at the primitive stage [11], we next examined the spatial organization of the PAC3K and PACΔ3K transgenes in embryonic blood samples. The restriction fragment containing 5′HS2 [24], a classical enhancer element within the LCR, was used as the fixed fragment for the results presented in Fig. 4. The analysis shows that the spatial organization of the locus is basically unchanged upon deletion of 5′HS5 (PACΔ3K) when compared with the PAC3K transgenic line. In agreement with the notion that the γ-globin gene is predominantly expressed in primitive cells, higher relative crosslinking frequencies of the *γ-globin* gene compared to the *β-globin* gene could be observed (Fig. 4). The results imply that in primitive erythroid cells association of the actively transcribed γ-globin genes with the ACH principally remains the same in the absence of 5′HS5.

**Figure 5:**
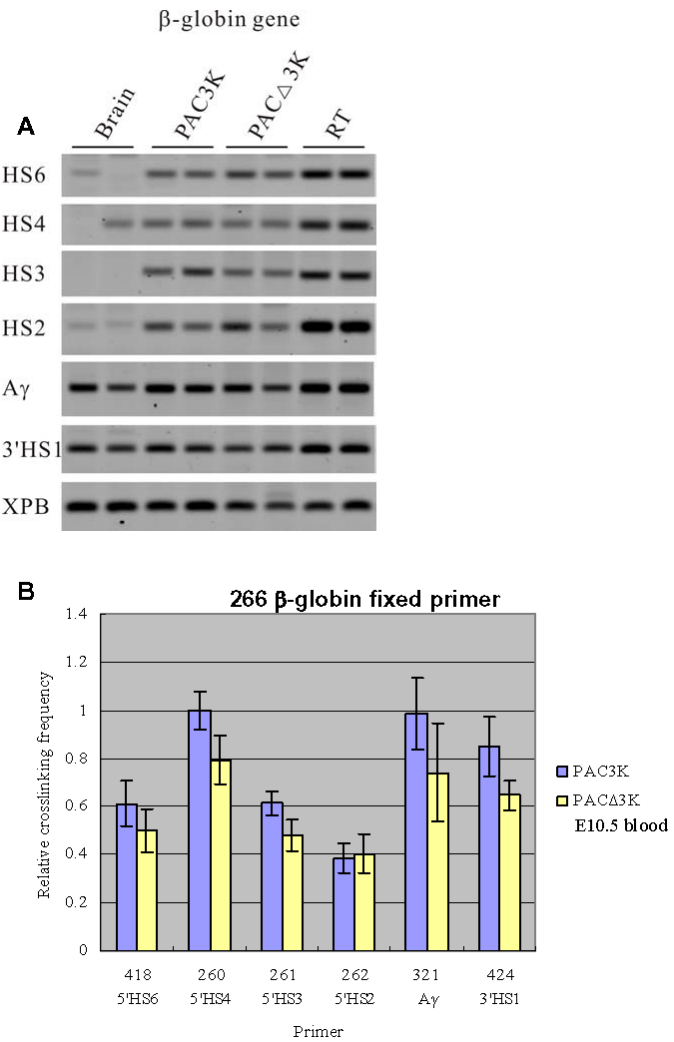
3C analysis of the PAC3K and PACΔ3K transgenes using a primer from the human *β*-globin gene as the fixed primer. Embryonic blood was collected from E10.5 embryo for this set of 3C experiment. (A) Representative expamples of the PCR fragments resulting from the 3C experiments. RT: Random template control. (B) Histograms of the relative crosslinking efficiencies after quantitation and normalization.

For investigating the spatial organization of the chromatin hub (CH), which can be observed in the erythroid progenitor cells without β-like globin gene expression [7], we used the *β-globin* gene as the fixed primer in 3C analysis (Fig. 5). Similar results were obtained between the two constructs (PAC3K vs. PACΔ3K). In conclusion, the results strongly indicate that deletion of 5′HS5 of the LCR in the human transgene does not have a measurable effect on the 3 dimensional organization of the *β-globin* locus, neither in the primitive stage where 5′HS5 functions as a border or enhancer blocker, nor in the definitive stage where 5′HS5 does not have a known function.

### ChIP analysis of CTCF binding to 5′HS5 in primitive and definitive erythroid cells

We have shown previously that human 5′HS5 has an enhancer blocking function at the primitive stage [11]. However, the mechanism by which this function is achieved remains unknown. Although a number of enhancer blocking proteins have been identified in *Drosophila* [25]–[27], CTCF is the only protein known so far to mediate enhancer blocking activity in vertebrates [28], [29]. Previous studies conducted by Felsenfeld and his colleagues have described a 1.2kb DNA element with strong enhancer blocking activity, located at the 5′HS4 of the chicken β-globin gene locus [28], [29].

A 250bp core fragment of the cHS4 is fully functional and a single binding site for the protein CTCF, FII, is necessary and sufficient to block the action of an enhancer on a promoter when placed between them [30]. Conserved CTCF binding sites are found at 5′HS5 and 3′HS1 of both the mouse and human loci.

**Figure 6:**
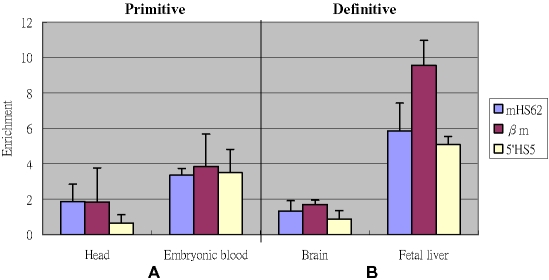
CTCF-ChIP assay analysis of the binding of CTCF protein to the mouse HS62 (mHS62), *β*m and human 5′HS5 in the primitive (A) and definitive (B) stage. Fold enrichment of the test sequence in bound with CTCF versus input/starting material is shown on the y axis.

To investigate whether CTCF binds to 5′HS5, ChIP analysis using an antibody to CTCF was performed (Fig. 6). The CTCF ChIP DNA enrichment levels of three fragments were tested, namely, mouse endogenous HS62 (mHS62), human βm (the marked β-globin gene) and human 5′HS5. In erythroid tissues, DNA enrichment could be detected by real-time PCR. Our results on mHS62 are in agreement with a previous study [14] and confirm that CTCF binds to the endogenous mouse HS62 fragment. Human 5′HS5 exhibits only modest enrichment levels of 1.5 fold at primitive and 2.4 fold at definitive stages. These results support the previous *in vitro* assay data [13] showing the presence of a CTCF binding site in human 5′HS5 [13]. Our data suggest that CTCF also binds to human HS5 *in vivo*.

Simultaneously, the βm gene enrichment level was determined using anti-CTCF ChIP assay. Surprisingly, the DNA enrichment level of βm gene is higher than that of the human 5′HS5 and mouse HS62 at both developmental stages (Fig. 6). Further studies are needed to characterize the functional role of CTCF binding to the βm gene.

## Discussion

In this study, we have analyzed the role of 5′HS5 with respect to the three dimensional configuration of the human β-globin locus. The transgenic lines carried PAC constructs that were derived from the original 185 kb human β-globin PAC [7]. Transgenic lines carrying single copies of four modified PAC constructs, namely PAC1B, PACΔ1B, PAC3K and PACΔ3K, were used [15] to determine possible conformational changes of the locus after deletion of the 5′HS5 core region. The 3D conformation of the transgenic loci was determined by 3C analysis. A human β-globin promoter deletion in the context of the full LCR was used to determine whether or not the interaction between the β-globin gene and the LCR would be perturbed when both 5′HS5 and the β-globin promoter (PACΔ3K) were deleted, as was previously found when both 5′HS3 and the β-globin promoter were deleted [23].

We analyzed the conformation of the globin transgenes from two important genomic sites in the human β-globin locus, namely the β-globin gene and 5′HS2. We first examined the integrity of the ACH upon the removal of 5′HS5 in the PACΔ1B transgenic line at the definitive stage. The results show that the three dimensional structure of the β-globin locus has essentially remained the same when 5′HS5 is deleted. Mild increments of the association frequency of 5′HS6 to the LCR could be explained by distance effects [9]: 5′HS6 interacts more frequently with the ACH once it moves closer to the LCR after Cre-mediated excision of 5′HS5. This is consistent with our previous data on human β-globin transgenic mice which showed that the increase in expression of the βm gene, which is located 5′ upstream of the LCR, is likely due to the shorter distance of the gene relative to the LCR [11].

Our previous study on the human β-globin PAC transgenes has shown that 5′HS5 is a stage-specific enhancer blocking element that functions at the primitive stage of erythropoiesis [11]. Surprisingly, we did not observe any significant alterations in the structure of the β-globin locus by 3C analysis primitive embryonic blood when 5′HS5 was deleted. The results obtained from both the definitive and primitive stages imply that spatial organization of the locus is essentially unchanged upon deletion of 5′HS5 (PACΔ1B) or in the double deletion of HS5 and the β-globin promoter (PACΔ3K). We can not completely discount the possible influence of 5′HS5 on β-globin locus configuration as this might not be detectable due to limitations of the resolution achieved with the 3C technique. However, we can conclude that the enhancer blocking properties of 5′HS5 in primitive erythroid cells is not accompanied by major effects on the spatial organization of the β-globin locus.

Interestingly this study shows that CTCF, the first transcription factor found to have insulating activity [28] and enhancer blocking function [13], [14] in mammalian cells, binds to the 5′HS5 core region at both the primitive and definitive stages in erythroid cells *in vivo*. This indicates that CTCF could be involved in the border function of 5′HS5 in the primitive cells. Since it also binds to the 5′HS5 region in definitive cells, this suggests that CTCF, most likely in conjunction with other proteins, can have opposing effects on gene expression. Filippova *et al.* [31] proposed that CTCF could be a transcriptional repressor of the *c-Myc* oncogene in vertebrates. In contrast, the CTCF protein can bind to the promoter of the amyloid β protein precursor and activate transcription [32]. Thus, CTCF could be a “dual functionality” protein that has divergent effects on different gene regulation systems. In addition, we show that CTCF binds to the βm gene in both primitive and definitive stage. Three putative CTCF binding sites can be identified at −133 to −149, −1005 to −990 and −1396 to −1412 bp relative to the cap site of the βm gene (Figure S1). Collectively, our results indicate that CTCF may have a more general role in the regulation of the β-globin locus than previously anticipated. Splinter *et al*. (2006) [33] have recently shown that in CTCF null cells, there were reduced DNA-DNA interaction frequencies between the sites that normally bind CTCF in the mouse β-globin gene locus. Interestingly, no changes were seen in the interaction of the surrounding olfactory genes (MOR5B1-3 and MOR3B1-4 [33]), or the expression level of the β-globin genes. It has been proposed previously that CTCF and the chick HS4 insulator sequence are highly concentrated in Matrix Attachment Regions (MARs) which create separate “loop” domains that prevent the interaction between an enhancer and promoter [34]. Collectively, these data suggested that CTCF participates in more than one pathway in the regulation of the globin genes.

## Materials and Methods

### Transgenic mice

PAC transgenic constructs (Fig. 1) containing the *β-globin* locus were generated using homologous recombination in *Escherichia coli* according to Iman *et al.* (2000) [15]. Modified PACs were used to generate transgenic mice; these mice have been characterized extensively in Wai *et al.* (2003) [11].

### Tissues used for 3C

Plugged transgenic mice were sacrificed and the embryos were collected at 10.5 and 14.5 dpc (day post coitum) respectively. Fetal liver and brain were collected from 14.5 dpc embryos, and embryonic blood and head were collected at 10.5 dpc. as previously described [6], [7], [35].

### Preparation of 3C templates

The 3C protocol was adapted from Tolhuis *et al.* (2002) [6]. 1×10^7^ cells was defined as 1 unit of sample in a 10ml crosslinking reaction (2% formaldehyde in DMEM with 10% FCS). The samples were cross-linked for 10 minutes at room temperature with gentle mixing. The reaction was quenched by the addition of glycine to 0.125M. Nuclei were harvested by lysis of the cells in 5ml ice-cold lysis buffer (10mM Tris-Cl, 10mM NaCl, 0.2% NP-40 pH8.0) containing protease inhibitors (pefabloc, Roche) for 10 minutes on ice. Nuclei were resuspended in 0.5ml 1× HindIII restriction buffer (Roche) containing 0.3% SDS and incubated for 1 hour at 37°C with agitation. Triton X-100 was added to 2% (v/v), and the nuclei were further incubated for 1 hour at 37°C to sequester the SDS. The crosslinked DNA was digested overnight at 37°C with 400U HindIII. The restriction enzyme was inactivated by the addition of SDS to 1.6% and incubated at 65°C for 20 minutes with agitation. The reaction was diluted with 7ml 1× ligation buffer (Promega) and incubated for 1 hour at 37°C. The chromatin was ligated with 100U T4 DNA ligase (Promega) for 4 hours at 16°C followed by 30 minutes at room temperature. 600 µg of Proteinase K was added, and samples were incubated overnight at 65°C to reverse the cross-links. Next, the samples were incubated for 30 minutes at 37°C with 1500 µg of DNase-free RNaseA, and the DNA was purified by phenol extraction and ethanol precipitation. The DNA pellet was resuspended in 150 µl 10 mM Tris-Cl (pH7.5).

### Preparation of Random Template control

In order to normalize the amplification efficiency of different primer sets, the PAC185 [15] plasmid containing the human *β-globin* locus was used for preparation of the random template control (RTC). Theoretically, this random template control contains all possible ligation products in equimolar amounts. Wild-type mouse genomic DNA was mixed with PAC185 plasmid and a 60–70kb PAC containing the mouse *XPB* locus (PAC Clone #443-C18, MRC gene services). The genomic DNA/PAC clones mixture was processed according to the 3C technique [6].

The processed products were serially diluted with wild-type mouse genomic DNA until the PAC DNA was present in a molar ratio similar to single-copy *β-globin* locus transgenic DNA (line PAC8.1), as assessed by quantitative PCR [6], [7]. PCR products were run on a 2% agarose gel and quantified with a Typhoon 9200 imager (Amersham). Typically 300ng of DNA was subsequently used for each 3C-PCR reaction. Each PCR reaction was performed in triplicate and repeated at least three times. Sequences of the 3C PCR primers are shown in Table 1.

**Table 1:** Sequence of 3C-PCR primers.

Target gene locus	Target sequence: primer #	Sequence (5′-3′)
Human β-globin	5′Olfactory Receptor gene:	318
		CAGCTTGGGTCAAATACAG
Human β-globin	Marked β-globin gene (βm):	474
		ACCACCCTTTCCACAGTC
Human β-globin	HS5:	442
		CTGCACACTTTCAGTCCG
Human β-globin	HS4	260
		CAAATGGGTGACTGTAGGG
Human β-globin	HS3	261
		GCCTGCATTTATTGTTGTG
Human β-globin	HS2	262
		GAACTGCTCATGCTTGGAC
Human β-globin	Aγ-globin gene (Aγ):	321
		GAAGAAAGACCTCTATAGGACAGG
Human β-globin	β-globin gene (β):	266
		TGGTTATGGTCAGAGCCTC
Human β-globin	3′HS1:	424
		ACATGGATAATACTGTTCCCC
Mouse XPB	XPB	341&343
	Forward:	TGACCTCCACACTCCTGAC
	Reverse:	ATGCGCAATTAGAAACTGC

### Anti-CTCF ChIP assays

1×10^7^ cells were defined as 1 unit of sample for 10ml ChIP crosslinking reaction (2% formaldehyde and 40mM HEPES pH7.9 in DMEM with 10% FCS). The samples were crosslinked for 20 minutes at room temperature with gentle mixing. The reaction was quenched by the addition of glycine to 0.125M. The cells were harvested by centrifugation at 1500rpm for 5 minutes at 4°C. The cells were first washed with PBS buffer containing protease inhibitors (1 mM phenylmethylsulfonyl fluoride (PMSF), 1 µg/ml aprotinin and 1 µg/ml pepstatin A) and centrifuged at 1500rpm for 5 minutes. 10 ml of Triton-wash buffer (0.25% Triton-X 100, 10mM Na-EDTA, 0.5 mM Na-EGTA and 10 mM Tris-Cl pH8.0) was added and the samples were incubated at room temperature for 10 minutes. The cells were pelleted by centrifugation at 1500rpm for 5 minutes and they were further washed with 10ml NaCl-wash (200mM NaCl, 1 mM Na-EDTA, 0.5mM Na-EGTA and 10mM Tris-Cl pH8.0) buffer at room temperature for 10 minutes. After centrifugation at 1500rpm for 5 minutes, the pellet was resuspended in 500 µl sonication buffer (1mM Na-EDTA, 0.5mM Na-EGTA and 10mM Tris-Cl pH8.0) and kept on ice for 5 minutes. DNA was sheared by sonication to lengths between 200 and 1000bp, keeping the sample cold on ice. Cell debris was removed by centrifugation. ChIP reactions were performed according to the Upstate Biotechnology chromatin immunoprecipitation protocol with 1 µg of anti-CTCF antibody (Upstate Biotechnology) per IP reaction. Enrichment of specific immunoprecipitated sequences, including 5′HS5, βm (marked *β-globin* gene), mHS62 (mouse HS62) and a fragment of the mouse *Necdin* gene (control), were determined by real-time PCR. Fold enrichment was calculated by comparing the ratio to *Necdin* in pull-down-eluate against the value from input-starting material. The sequences of the ChIP PCR primers are shown in Table 2. The location of the βm gene primers were shown in Fig. 1B.

**Table 2:** PCR primers used in the Anti-CTCF ChIP Real-time PCR experiments.

Target sequence: primer name	Sequence (5′-3′)	Amplicon length (bp)
ChIP-βm		
Forward (βm-F):	TCCTAAGCCAGTGCCAGAAGAG	236
Reverse (βm-R):	TGTCCTCCTCTGTGAAATGAC	
ChIP-HS5		
Forward:	TAGCTGAAGCTGCTGTTATGACCAC	149
Reverse:	CCAGATGTCCTGTCCCTGTAAGGT	
ChIP-mHS62		
Forward:	TGCCGTAGTTCTCTAGTGTAGCCAC	170
Reverse:	TATGGGGTGTGGGTATTTGTAAGAG	
ChIP-necdin		
Forward:	AGTTCTGTGCCATACAGGAGAC	212
Reverse:	AGAGGAAGTGCGCTTTACTGAG	

## Supporting Information

Figure S1 Alignment of the cHS4 CTCF binding site with the βm promoter. Alignment of the cHS4 CTCF binding site with the βm promoter (1). The βm gene cap site (+1) are indicated with a blue letters. The proposed CTCF binding sites in the βm sequence are indicated with red letters and the cHS4 CTCF binding sequence is shown on top of each proposed binding site. Conserved binding sequences are highlighted with a gray background. The alignment was performed with the ClustalX program (2) Reference List 1. Farrell CM, West AG, Felsenfeld G (2002) Conserved CTCF insulator elements flank the mouse and human beta-globin loci. Mol Cell Biol 22: 3820-3831. 2. Thompson JD, Gibson TJ, Plewniak F, Jeanmougin F, Higgins DG (1997) The CLUSTAL_X windows interface: flexible strategies for multiple sequence alignment aided by quality analysis tools. Nucleic Acids Res 25: 4876-4882.
(0.04 MB DOC)
