# Supplementary material for: 5′HS5 of the Human β-globin Locus Control Region Is Dispensable for the Formation of the β-globin Active Chromatin Hub
Source: PLoS One. 2008 May 7;3(5):e2134. doi: 10.1371/journal.pone.0002134 (PMC2358975; doi:10.1371/journal.pone.0002134)
Supplement: Figure S1 — Alignment of the cHS4 CTCF binding site with the βm promoter. Alignment of the cHS4 CTCF binding site with the βm promoter (1). The βm gene cap site (+1) are indicated with a blue letters. The proposed CTCF binding sites in the βm sequence are indicated with red letters and the cHS4 CTCF binding sequence is shown on top of each proposed binding site. Conserved binding sequences are highlighted with a gray background. The alignment was performed with the ClustalX program (2) Reference List 1. Farrell CM, West AG, Felsenfeld G (2002) Conserved CTCF insulator elements flank the mouse and human beta-globin loci. Mol Cell Biol 22: 3820-3831. 2. Thompson JD, Gibson TJ, Plewniak F, Jeanmougin F, Higgins DG (1997) The CLUSTAL_X windows interface: flexible strategies for multiple sequence alignment aided by quality analysis tools. Nucleic Acids Res 25: 4876-4882. (0.04 MB DOC) [file pone.0002134.s001.doc]

aagaaatttgtaaatttccttctgataactagaaatagaggatccagttt

cttttggttaacctaaattttatttcattttattgttttattttatttta

aaaaaaaaaaaaaaaaaaaaaaaaaaaaaaaaaaaaaacca-ctagaggg

ttttattttattttgtgtaatcgtagtttcagagtgttagagctgaaagg

aagaa

aagaagtaggagaaacatgcaaagtaaaagtataacactttccttactaa

accgactgggtttccaggtaggggcaggattcaggatgactgacagggcc

cttagggaacactgagaccctacgctgacctcataaatgcttgctacctt

tgctgttttaattacatcttttaatagcaggaagcagaactctgcacttc

aaaagtttttcctcacctgaggagttaatttagtacaaggggaaaaagta

cagggggatgggagaaaggcgatcacgttgggaagctatagagaaagaag

agtaaattttagtaaaggaggtttaaacaaacaaaatataaagagaaata

aaaaaaaaaaaaaaaaaaaaaaaaaaaaaaaaaaaaaaaaaaaaaccact

ggaacttgaatcaaggaaatgattttaaaacgcagtattcttagtggact

agagggaagaa

agaggaaaaaaataatctgagccaagtagaagaccttttcccctcctacc

cctactttctaagtcacagaggctttttgttcccccagacactcttgcag

attagtccaggcagaaacagttagatgtccccagttaacctcctatttga

caccactgattaccccattgatagtcacactttgggttgtaagtgacttt

ttatttatttgtatttttgactgcattaagaggtctctagttttttatct

cttgtttcccaaaacctaataagtaactaatgcacagagcacattgattt

gtatttattctatttttagacataatttattagcatgcatgagcaaatta

agaaaaacaacaacaaatgaatgcatatatatgtatatgtatgtgtgtat

atatacacatatatatatatattttttttcttttcttaccagaaggtttt

aatccaaataaggagaagatatgcttagaactgaggtagagttttcatcc

attctgtcctgtaagtattttgcatattctggagacgcaggaagagatcc

atctacatatcccaaagctgaattatggtagacaaagctcttccactttt

agtgcatcaatttcttatttgtgtaataagaaaattgggaaaacgatctt

caatatgcttaccaagctgtgattccaaatattacgtaaatacacttgca

aaggaggatgtttttagtagcaatttgtactgatggtatggggccaagag

atatatcttagagggagggctgagggtttgaagtccaactcctaagccag

aaccactagagggaagaa

tgccagaagagccaaggacaggtacggctgtcatcacttagacctcaccc

tgtggagccacaccctagggttggccaatctactcccaggagcagggagg

gcaggagccagggctgggcataaaagtcagggcagagccatctattgctt

aca

-1501

-1451

-1401

-1351

-1301

-1251

-1201

-1151

-1101

-1051

-1001

-901

-851

-801

-751

-701

-651

-601

-551

-501

-451

-401

-351

-301

-251

-201

-151

-101

-51

-1

+1
